# Effect of Source Type and Protective Message on the Critical Evaluation of News Messages on Facebook: Randomized Controlled Trial in the Netherlands

**DOI:** 10.2196/27945

**Published:** 2022-03-31

**Authors:** Frans Folkvord, Freek Snelting, Doeschka Anschutz, Tilo Hartmann, Alexandra Theben, Laura Gunderson, Ivar Vermeulen, Francisco Lupiáñez-Villanueva

**Affiliations:** 1 Open Evidence Research Open Evidence Barcelona Spain; 2 Nijmegen Netherlands; 3 Behavioural Science Institute Radboud University Nijmegen Netherlands; 4 Communication Science Vrije Universiteit Amsterdam Netherlands; 5 Barcelona school of management UPF Barcelona School of Management Barcelona Spain

**Keywords:** health communication, disinformation, protective message, source, critical evaluation, critical thinking

## Abstract

**Background:**

Disinformation has become an increasing societal concern, especially due to the speed that news is shared in the digital era. In particular, disinformation in the health care sector can lead to serious casualties, as the current COVID-19 crisis clearly shows.

**Objective:**

The main aim of this study was to experimentally examine the effects of information about the source and a protective warning message on users’ critical evaluation of news items, as well as the perception of accuracy of the news item.

**Methods:**

A 3 (unreliable vs reliable vs no identified source) × 2 (with protective message vs without) between-subject design was conducted among 307 participants (mean age 29 (SD 10.9] years).

**Results:**

The results showed a significant effect of source information on critical evaluation. In addition, including a protective message did not significantly affect critical evaluation. The results showed no interaction between type of source and protective message on critical evaluation.

**Conclusions:**

Based on these results, it is questionable whether including protective messages to improve critical evaluation is a way to move forward and improve critical evaluation of health-related news items, although effective methodologies to tackle the spread of disinformation are highly needed.

**Trial Registration:**

ClinicalTrials.gov NCT05030883; https://clinicaltrials.gov/ct2/show/NCT05030883

## Introduction

### Background

Disinformation is an increasing societal concern in many countries and had been highly apparent during the COVID-19 crisis [1-5]. Following the definition established by UNESCO (United Nations Educational, Scientific and Cultural Organization) [4], disinformation is considered in this study as information that is false and deliberately created to harm a person, social group organization, or country [4-6], while misinformation is defined as information that is false but has not been created with the intention to harm someone and malinformation is defined as information based on reality and used to inflict harm on a person, social group, organization, or country. During times of crises, such as the current COVID-19 situation, disinformation in the health space is thriving. Therefore, European institutions are jointly fighting against disinformation and working in close cooperation with online platforms to ensure effective public health communication [1,7,8].

Transparency regarding the way information is produced or sponsored is necessary to support high quality (digital) information provision, and the relation between information creators and distributors needs some rebalancing [9]. Disinformation may lead to inaccurate beliefs and can exacerbate partisan disagreement over basic facts that could easily be checked [10]. There may have been instances of dissemination of false or misleading information in the past, but in contemporary societies its consequences arrive faster, become more visible, and therefore seem to harm current societies to a larger extent than before. In particular, Facebook was used as a platform to direct users to websites where disinformation was published, showing the importance of the hierarchical, institutional, or professional structures that traditional media provides as a gatekeeper but social media platforms miss because of their origin [11,12].

### Literature Review

Following the truth-default theory [13], people generally tend to believe others as a default state, a so-called “truth bias.” Levine [13] proposes that this truth default is adaptive, based on contextual and informational factors. Considering that belief in honesty is a default state, which is a passive starting place for making inferences about communication, the truth-default theory predicts that once suspicion is actively triggered, the possibility of processing a message as deception might occur.

Nowadays, social media platforms have become de facto news curators [14,15]. Since many people, particularly youth [3], consume their main news information from social media platforms, the influence of disinformation has become a real threat around the world and should be investigated [16-19]. For example, about half of American adults (53%) report that they get news from social media often or sometimes [17].

Due to the information flows provided by the internet, information today can be published by anyone and come from anywhere, which is a participatory strength of an open society but also has its weaknesses [20,21]. Disinformation often contains appealing titles and salient pictures that cue emotional arousal, giving rise to impulsive sharing decisions [22]. Consequently, disinformation is often quickly shared on social media after the sharer has read the attractive title or appealing highlights but perhaps not the complete content [23].

### Empirical Evidence

Regardless of why the information was shared, this phenomenon only reinforces the problem of the dissemination of disinformation [20]. Vosoughi et al [24] demonstrate that the rate of spreading of disinformation is 6 times that of spreading truthful content. Bode and Vraga [25,26] suggest that once disinformation is absorbed, it is much more difficult to change the misperception or belief (ie, rectify it) and that it can take a long time to resolve false rumors [27]. In particular, youths find it more difficult to overcome the spread of disinformation, as they are digital natives and most fake news is shared on online platforms [28]. Another study showed university students visiting social media platforms more often, and sharing and liking posts increases the susceptibility of students to fake news [29]. Furthermore, Buchanan showed that sharing false information online was not influenced by authoritativeness of the source of the material, and participants’ level of digital literacy had little effect on their responses [30]. Importantly, multimodal disinformation, which occurs mainly on social media, is considered slightly more credible than textual disinformation [31].

A meta-analysis by Chan et al [32] shows that media literacy can affect the extent to which people can interpret disinformation. The researchers recommend improving people’s media literacy to encourage a critical attitude toward the processing of disinformation. Media literacy allows users to be more critical of what they see and read, so they can judge for themselves whether they believe the information [32]. More specifically, eHealth literacy, the competence of people to accurately comprehend health information, also influences individual susceptibility to health-related information provided on social media [33].

In addition, recent research by Clayton et al [34] shows that disinformation on Facebook is perceived to be less accurate when a protective warning message is posted announcing that disinformation occurs on this platform. The authors argue that more research needs to be done to ensure disinformation is effectively combated to reduce the negative effects on health care provision, but that real and accurate news does not suffer from the effects of such an intervention [34]. Therefore, it seems important to focus on stimulating critical processing of the content of news messages rather than just using a warning that influences the perception of accuracy of news messages in general. Unfortunately, relatively little is known about effective interventions that cause Facebook users to evaluate information more critically, thus enabling them to recognize disinformation more easily.

### Study Objective and Hypotheses

**Figure 1:**
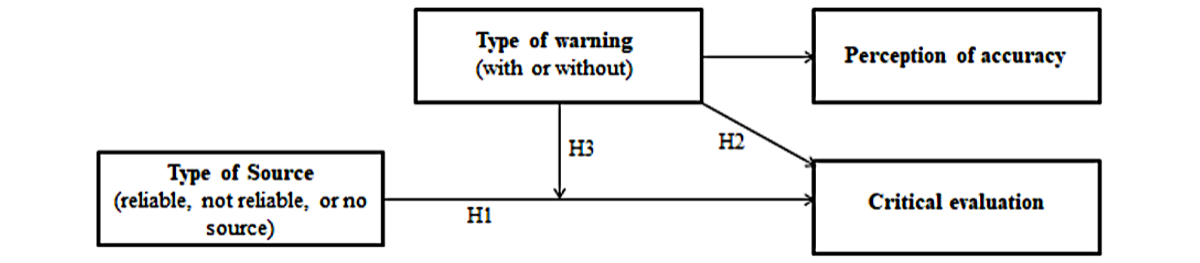
Conceptual model.

The main objective of this study is to gain more insight into the extent to which source information and a protective message have an effect on critical evaluation by Dutch Facebook users. In Figure 1 we show the conceptual model. Based on the truth-default theory, the expectation is that participants exposed to news messages from an unreliable source will activate more critical reflection than participants exposed to news messages from a reliable or unidentified source (H1a). Furthermore, it is hypothesized that participants exposed to news messages from an unreliable source will perceive these news items as less accurate than participants exposed to news messages from a reliable or unidentified source (H1b). In addition, participants exposed to news messages following a protective message will activate more critical reflection (H2a) and perceive these items as less accurate (H2b) than participants exposed to news messages without a preceding protective message. Finally, it is expected that adding a protective message will moderate the effect of the credibility of the source on critical reflection and perceived accuracy of news items. More specifically, we expect that participants exposed to news messages from an unreliable source who have previously encountered a protective message will activate more critical reflection (H3a) and perceive news items as less accurate (H3b) than participants who have not encountered a protective message before. In contrast, we do not expect any difference in critical reflection and perceived accuracy to be instigated by the protective message among participants exposed to news messages from a reliable or unidentified source.

## Methods

### Design

To test the hypotheses and investigate the effects of source information and a protective message on critical evaluation and perception of accuracy of news items on Facebook, an online between-subjects experiment with 3 (unreliable vs reliable vs unidentified source) × 2 (with protective message vs without) levels was constructed. Six conditions were created in line with the factorial design of the study, and participants were randomly assigned to one of the conditions. Participants’ critical evaluation and the perception of accuracy of the presented (news) messages were assessed as dependent variables.

### Stimulus Material

The stimulus material consisted of a short video clip showing how an (anonymous) user opens the Facebook app on a smartphone to scroll over the Facebook timeline. Considering that many people consume their main news from social media [17], we decided to use Facebook as stimulus material. This is a daily activity for many Facebook users, and the video clip aimed to imitate this daily activity as realistically as possible. The Facebook timeline featured in the clip contained 3 short (news) messages shown one after the other. Each (news) message was on screen for 10 seconds. No other messages were shown in the video.

The (news) messages were about ambiguous health-related topics so it would not be immediately apparent to participants whether the news messages was accurate [11]. This setup made it possible to test the effects of the source type and protective message. The presented (news) messages addressed vaccination [35], climate change [36], and health insurance [37]. The (news) messages about vaccination and health insurance were made up for this study, and the climate report was a real news report from a Dutch public broadcaster, Nederlands Omroep Stichting (NOS) [38]. The headline for the vaccination item was “Prohibition for unvaccinated children” and the body text was “Is your child not vaccinated? Then he or she is no longer welcome at daycare centers and primary schools. This decision made last Monday. There is an unnecessarily high risk and that is why this ban is necessary, the government says.” The headline for the climate change item was “Dutch aviation’s impact on climate change is going to be enormous” and the body text was “If no new measured are taken, the CO_2_ emissions of Dutch aviation will double over the next 30 years. This will make aviation one of the largest polluters in the Netherlands in 2050.” The headline for the health insurance item was “Deductible amount in health care will double next year” and the body text was “Health insurers can no longer finance high health care costs if people’s deductible amount remains so low. Deductible amount must increase considerably in order to be able to provide good care for everyone.”

### Source Information

To examine the influence of source information, both above and below each news message an unreliable, reliable, or unidentified source was provided. The choice of the unreliable source was based on previous research by Pennycook and Rand [39], who used the existing news site Dailybuzzlive to represent an unreliable source. The reliable source chosen was NOS, based on research by the Media Monitor [40] showing that NOS is perceived as the most reliable news source in the Netherlands. Unidentified source (news) messages were shown without source information.

### Protective Message

**Figure 2:**
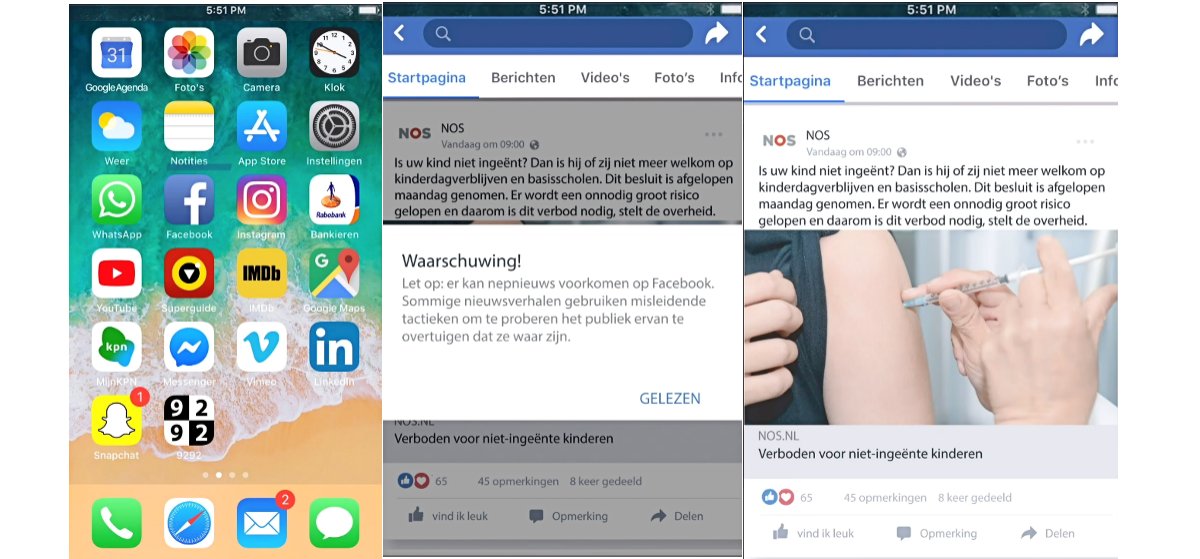
Stimulus material.

To examine the influence of a protective message, half of the participants received a protective message in the video displaying the (news) messages. After the anonymous users displayed in the video opened the Facebook app, a warning about fake news appeared. This warning was in Dutch and motivated by previous research by Clayton et al [34]. It read “Warning! Note: fake news can occur on Facebook. Some news stories use misleading tactics to try and convince the public that they are true.” The warning was on the screen for 10 seconds, so the short video clip with a warning lasted 10 seconds longer (48 seconds in total) than the clip without a warning. Figure 2 shows an example of how a reliable source with a protective message was presented.

### Participants

**Figure 3:**
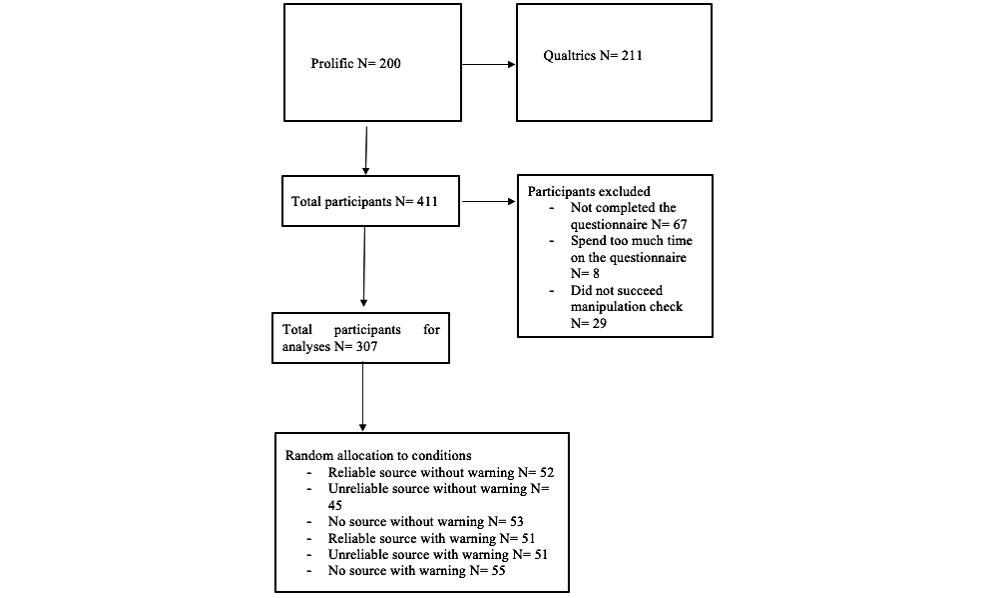
Flow diagram of study participants.

Participants were recruited during May and June 2019 through an online panel (Prolific) and a Qualtrics link shared on social media channels such as Facebook, Instagram, and LinkedIn. Analyses were conducted separately for both groups of participants, but no different effects were found. Therefore, we have decided to aggregate the two groups and only report the analyses for the complete group of participants. A G*power analysis (effect size=0.25, alpha=.05, power=.80, and two predictors) determined a minimum sample size of 158 participants [41]. In total, 411 participants were recruited to the study, however, 67 participants did not fully complete the questionnaire. In the experiment, it was important that participants completed the questionnaire without pausing because this could interrupt the flow and would harm the validation of the protective message. In order for the effect of the manipulation to be tested accurately, participants (n=8) who needed more than 15 minutes to complete the questionnaire were removed. The control question was a check to see if participants paid attention to the stimulus material, and participants (n=29) who could not remember what the news items were about (assessed with a closed question) were removed. After dropping participants who did not meet the abovementioned standards, the total number of participants was 307 (see Figure 3).

### Ethics Approval

The ethical committee of the Tilburg School of Humanities and Digital Sciences (REDC 2019-60, obtained April 29, 2019) in the Netherlands approved this study. This trial was registered at ClinicalTrials.gov [NCT05030883].

### Procedure

Participants conducted the experiment in an online survey in Prolific and Qualtrics that included a random allocation procedure. Furthermore, the participants participated anonymously, and informed consent was provided by the participants. Next, participants were asked to report whether they had a Facebook account, and sociodemographic data were collected (gender, age, education level). Next, participants were randomly allocated to one of the 6 experimental conditions. After the video clip, users’ general critical evaluation of the 3 displayed (news) messages was assessed. This was followed by questions about the perception of accuracy. Additional questions were then asked, such as reasons for Facebook use, frequency of Facebook use, whether participants follow the news via Facebook, whether they trust the news on Facebook, and to what extent they generally follow the news. Finally, a manipulation check was performed. People were asked if participants had seen a warning and what this warning was about and if they had observed a news source and what news source it was. After the last question a debriefing explained the purpose of the research. After the debriefing, participants were thanked for their participation.

### Measurements

#### Dependent Variables

##### Critical Evaluation Skills

Critical evaluation was assessed based on a scale developed by Metzger et al [41]. This unidimensional scale consists of 9 items on 5 different aspects (accuracy, authority, objectivity, currency, and coverage), testing on a 5-point Likert scale (1=never, 5=always) if participants conducted these behaviors when reading news (Table 1). To confirm the unidimensional structure reported by Metzger et al [41], a principal component analysis (PCA) with oblimin rotation was performed across the 9 variables. We decided to use PCA over confirmatory factor analysis because we were interested in all the variances of the data we collected. The Kaiser-Meyer-Olkin test showed that it was an adequate sample to perform a PCA, sample adequacy is .91 (great according to Field [41]), Bartlett test of sphericity was significant (*χ*^2^_36_=1573.62, *P*<.001), and all variable loadings were >.606. The analysis yielded a single factor (Eigenvalue 5.15) that explained 57.28% of the total variance. Factor loadings are shown in Table 1. Hence, all 9 items were collapsed into a mean index, Cronbach *α*=.90, mean 3.15 [SD .93].

**Table 1:** Principal component analysis critical evaluation.

Item	Component loading
Check if the information is up to date	0.730
Check the source	0.820
Verify the quality of the source	0.871
Check the origin of the source	0.818
Find out if the source is officially recognized	0.774
Consider whether the information presented is fact or opinion	0.739
Find out or try to understand the intent of the author	0.606
Find other sources to validate the information	0.625
Verify that the information is complete and comprehensive	0.786

##### Perception of Accuracy

This variable is based on earlier research by Clayton et al [34] and consisted of 1 question per (news) message. The aim of the question was to find out what perception of accuracy participants had with a (news) message. The participant was asked to what extent they thought the statements in the (news) message were accurate. This was measured on a 4-point Likert scale (1=not at all accurate, 4=very accurate).

#### Control Variables

Multiple variables were assessed as potential covariates. For example, participants were asked how often they use Facebook (1=daily, 7=never; based on Clayton et al [34]), whether they followed the news via Facebook (1=always, 5=never; based on the Pew Research Center [3]), and if they had confidence in news on Facebook (1=completely disagree, 5=completely agree) [41]. In addition, a second statement asked whether participants followed the news on a daily basis (1=completely disagree, 5=completely agree) [3]. Finally, participants were asked their gender (male/female), age (numerical), and the highest level of education completed.

#### Manipulation Check

Participants answered 4 questions at the end of the experiment to check whether manipulation was observed. First, they were asked if they had seen a warning in the video. Participants could answer this question with yes or no. When participants had seen a warning, they were then asked what the warning was about. There were 4 choices: internet limit, fake news, virus, and changes in news. Second, participants were asked whether they had seen a news source in the video. Here too, participants were able to answer yes or no. When participants had seen a news source, they were asked which news source they had seen. They could choose from Volkskrant, Dailybuzzlive, NOS, NederlandsNieuws, and I don’t remember.

## Results

### Descriptive Information

Study participants were 53.7% (165/307) men and 46.3% (142/307) women. The average age of the participants was 29 [SD 10.90] years. The highest completed level of education of participants was mainly higher educated, with 0.3% (9/307) reporting primary school, primary education, or no education as highest educational level; 1.3% (40/307) reporting preparatory secondary vocational education or lower general secondary education; 15.0% (46/307) reporting school of higher general secondary education or the preuniversity education; 7.5% (23/307) reporting secondary vocational education; 37.8% (116/307) reporting higher professional education; and 38.1% (117/307) reporting university. All participants had a Facebook account. Participants mainly used Facebook to stay in touch with friends and family (221/307, 72.0%) and to stay informed about events (148/307, 48.2%). In addition, 25.7% (79/307) of participants indicated that they used Facebook for news and information, 20.5% (63/307) for pastimes, and 12.4% (38/307) to be able to follow brands or famous people. On average, participants spend a few times a week on Facebook (mean 2.08 [SD 1.50]) and on average they occasionally followed the news via Facebook (mean 3.74 [SD 1.03]). In addition, confidence in news via Facebook was low (mean 2.28 [SD .91]) and participants on average followed the news daily (mean 3.68 [SD 1.16]).

### Correlational Analyses

To test for potential covariates, Pearson correlations were calculated between all control variables and critical evaluation and perceived accuracy of the news item. Only “trust in news via Facebook” (*r*=–.140, *P*=.01) and “the daily following of news” (*r*=.208, *P*<.001) were significantly related to critical evaluation. Participants with lower trust in Facebook news and greater daily news consumption engaged in more critical evaluation of the presented (news) messages. Therefore, “trust in news via Facebook” and “daily monitoring of news” were included as covariates in the analyses.

### Manipulation Checks

First, a manipulation check on the source information showed that 88.3% (91/103) of participants in the conditions with a reliable source saw a news source, while 11.7% (12/103) did not. In conditions with an unreliable source, 63% (60/96) of participants indicated that they had seen a news source, and 38% (36/96) did not. In the conditions without a news source, 64.8% (69/108) indicated that they had not seen a news source and 36% (39/108) indicated that they had seen a news source. The chi-square test (*χ*^2^_2_=61.02, *P*<.001) showed that the differences between the experimental conditions, whether or not participants saw a news source, were significant. This means that the manipulation, whether participants have seen a news source or not, has been successful. Nonetheless, we tested the hypotheses with and without the noncompliers. We found no different in the results with and without noncompliers. Therefore, we decided to include all in the final analysis.

Second, of the participants who have seen a reliable news source, 96.7% (88/91) named NOS.nl as a source. In the unreliable source condition, 95% (57/60) indicated that they had seen Dailybuzzlive.nl. A salient feature is that 36.1% (39/108) of participants in the no source condition indicated that they had seen a certain news source, but this was not the case. For example, 7 participants indicated that they had seen Volkskrant.nl, 6 participants Dailybuzzlive.nl, 15 participants NOS.nl, 5 participants NederlandsNieuws.nl, and 6 participants could not remember.

Third, when asked whether participants had seen a warning in the video on Facebook, in the conditions without a warning, 92.7% (139/150) of participants indicated that they had not seen a warning. The remaining participants (11/150, 7.3%) indicated that they had seen a warning. In the conditions with a protective message, 91.1% (143/157) of participants indicated that they had seen a warning and 8.9% (14/157) indicated that they had not seen a warning. The chi-square test (*χ*^2^_1_=215.22, *P*<.001) showed that the differences between the experimental conditions, whether participants had seen a warning or not, were significant. From this it can be concluded that the manipulation was successful. Of the participants who saw a protective message, 97.2% (139/143) indicated that the warning was about fake news.

### Testing of the Hypotheses

An analysis of covariance (ANCOVA) on critical evaluation showed a significant main effect of source information on critical evaluation (F_2,299_=6.401, *P*=.02). Post hoc analysis with Bonferroni correction showed that participants exposed to a news message with an unreliable source (mean 3.31, SE .09) scored significantly higher (*P*=.02) on critical evaluation than the group with a reliable source (mean 2.95, SE .09). There was no significant difference between no source (mean 3.19, SE .09) and a reliable (*P*=.18) or unreliable source (*P*>.99). These results partly support H1a. Furthermore, the ANCOVA showed that there is a marginally significant relation between trust in news via Facebook and critical evaluation (F_1,299_=3.62, *P*=.06) and a significant relation between following the news daily and critical evaluation (F_1,299_=15.14, *P*<.001).

In addition, the ANCOVA showed that a protective message did not significantly affect critical evaluation (F_1,299_=1.59, *P*=.21), thereby rejecting H2a. Finally, the ANCOVA showed that there was no interaction effect of type of source and protective message on critical evaluation (F_2,299_=0.41, *P*=.67), thereby rejecting H3a.

Next, we tested whether a protective message had an influence on the perception of accuracy in (news) messages on Facebook. A multivariate analysis of covariance (MANCOVA) analysis showed that there was no significant difference between groups that had seen a protective message and groups that had not on the perception of accuracy (Wilks Λ=0.998, F_3,303_=0.24, *P*=.87), thereby rejecting H1b. Univariate tests showed that these groups did not differ on the (news) message about vaccination (F_1,305_=0.08, *P*=.77, *η*^2^<.001), climate change (F_1,305_=0.72, *P*=.40, *η*^2^=.002), and health insurance (F_1,305_=0.07, *P*=.79, *η*^2^<.001). This means that a protective message has no effect on the perception of accuracy.

Further, the MANCOVA analysis showed that there was a significant difference between source information on the perception of accuracy (Wilks Λ=0.929, F_2,304_=3.79, *P*<.001). The outcomes of univariate tests show that the perception of accuracy by source information in the (news) message differed significantly for vaccination (F_2,304_=8.02, *P*<.001, *η*^2^=.050) and health insurance (F_2,304_=4.01, *P*=.02, *η*^2^=.026). There was no significant effect on the (news) message about climate (F_2,304_=2.34, *P*=.10, *η*^2^=.015). These partly confirm H2b.

To see which conditions of source information differed significantly from each other on perception of accuracy, a post hoc analysis with Bonferroni correction was performed. The results showed that there was a significant difference between a reliable and an unreliable source. The post hoc analysis showed that the group with a reliable source (mean 2.85, SE .08) scored significantly higher in the (news) message about vaccination (*P*<.001) on perception of accuracy than the group with an unreliable source (mean 2.39, SE .09). There was a marginally significant difference between no source (mean 2.58, SE .08) and a reliable source (*P*=.06) and no significant difference between no source and an unreliable source (*P*=.27). There was also an effect on the (news) message about health insurance. The group with a reliable source (mean 2.33, SE .08) scored significantly higher (*P*=.04) on perception of accuracy than the group with an unreliable source (mean 2.03, SE .08). There was a marginally significant difference between no source (mean 2.06, SE .08) and a reliable source (*P*=.05) and no significant difference between no source and an unreliable source (*P*>.99). This means that source information has an effect on the perception of accuracy in the (news) messages about vaccination and health insurance.

Finally, the MANCOVA showed that there was no interaction effect on type of source and protective message on perceived accuracy of all 3 messages (*P*>.05), thereby rejecting H3b.

## Discussion

### Principal Findings

The main objective of this study was to gain insight into the extent to which source information and a protective message have an effect on the critical evaluation by Dutch Facebook users. This study showed that source information has an effect on the extent to which someone critically evaluates (news) messages on Facebook. In accordance with the first hypothesis, participants more critically evaluated a (news) message when exposed to an unreliable source compared to a reliable source. No significant differences were found between an unreliable or reliable source and an unidentified source. In accordance with Metzger et al [41], the findings of this study also imply that participants use cognitive heuristics to assess information by critically evaluating to a greater or lesser extent. Source credibility as a heuristic seems to play a determining role in the extent to which someone tends to critically evaluate a (news) message on Facebook [42,43].

In addition, we expected that a protective message preceding disinformation on Facebook would have a positive effect on the critical evaluation of participants. The truth-default theory [13] predicted that once suspicion was actively triggered, the possibility of processing a message as deception might come to mind. No evidence for such an effect was found, which is why hypothesis 2 was rejected. In addition, no evidence was found that a protective message moderated the effect of source information on critical evaluation. This, therefore, rejected hypothesis 3. Current solutions provided by the European Commission and large social media platforms promising to include protective messages to improve critical evaluation to tackle the spread of disinformation may therefore be of only limited effectiveness, since most people believed the message was still valid and credible. This study showed that the inclusion of protective measures of this type might, in fact, not affect critical evaluation.

Despite positive results of a protective message found by others [2,34], we did not find any effect of a protective message on critically processing news information. A possible explanation for this is that participants’ flow while reading the Facebook news had not been interrupted and, therefore, they had not processed the protective message with sufficient attention [44]. In addition, it is possible that people were not motivated or involved enough to critically evaluate the news messages on Facebook [45]. A suggestion for a follow-up study is that participants are obligated to look at the protective message and need to click on the protective message to continue, stopping their current activity and attention for the task they are conducting and activating cognitive resources to process the protective message.

A protective message was expected to reinforce the effect of source information on critical evaluation, which would result in participants evaluating more critically when exposed to an unreliable source. No evidence for this was found. It seems that source information has a greater influence on the critical evaluation of people than a protective message. Perhaps participants were already familiar with the fact that news on Facebook might be unreliable (see also the relatively low score on trust in Facebook news), which made them less involved in processing this message and instead made other cues that give more specific (source) information about whether an actual news message is reliable more important. This might imply that acknowledgement of the source is a more promising intervention when stimulating critical processing is the aim, although more research is needed to verify this.

The second aim of this study was to investigate whether a protective message had an effect on the perception of accuracy in (news) messages on Facebook. Here, the results also show that a protective message has no significant effect. What is striking is that an additional analysis shows that source information can have an undesirable effect on the perception of accuracy of (news) messages that are fake. When disinformation was shown with a reliable source, the news was considered more accurate than when the disinformation is shown with an unreliable source. The source, thereby, functions as a heuristic for accurate news, while in actuality disinformation is distributed. This study showed that an unreliable source, compared to a reliable source, can cause users to evaluate (news) messages on Facebook more critically. An explanation for this is that people process the reliable source more easily and then trust it more quickly, especially in the social media context that mostly involves quick and casual reading. This results in participants believing a news message from a reliable source more quickly [42,43].

### Limitations

This research has several strengths. First, this research makes an important contribution to the social debate about how to deal with online disinformation, by providing scientific evidence on a large and important target group. Second, the stimulus material—a video—attempts to simulate a natural situation on Facebook. The video showed a Facebook timeline with 3 short news messages, comparable to actual news consumption among a large part of the population, increasing the ecological validity of this study. Nonetheless, this research also has a number of limitations. A first limitation of this research is that participants were not asked about the perception or reliability of the chosen news sources. It is unknown whether the participants perceived NOS.nl as a reliable news source, although previous reports have shown this [40], and during the COVID-19 crisis, millions of Dutch people used information from NOS as a source on new developments. A second limitation is that Dailybuzzlive.nl is not characterized by all participants as a news source. Participants may have processed the manipulation but did not interpret Dailybuzzlive.nl as a news source, although this also occurs in real life, where people read items on websites but do not directly consider them as news sources. Third, we only tested the experiment in the Netherlands, so it is difficult to generalize to other countries, particularly in countries where media literacy or eHealth literacy is not high. Considering the importance of the study, this study should be replicated in other countries to see if the outcomes remain the same. Fourth, although our sample size was sufficiently large to detect main effects of news source and warning message (sensitive to detect effect sizes larger than *η*^2^=.025 with .80 power), it may have been limited in its capability to detect interaction effects; interaction effect sizes are often smaller than main effect sizes and therefore are harder to detect [46].

### Conclusions

This study contributes to the social and scientific debate about how to treat online disinformation. The research shows that a simple intervention involving the acknowledgement of the source of a (news) message can have a positive effect on the critical evaluation by Facebook users. Furthermore, a protective message does not seem to contribute to the battle against online disinformation. A protective message had no effect on critical evaluation by users and no effect on the perception of accuracy. The results of this research serve as advice for Facebook to show the source more prominently so that users can quickly and easily see where a (news) message is coming from. Source information also has an effect on the perception of accuracy. Strikingly, participants regarded disinformation as more accurate when it came from a reliable news source. This means that reliable news sources, such as the NOS, have a responsibility not to disseminate disinformation, since people are more likely to believe this.

A recommendation for further research is to conduct follow-up studies on source information on Facebook or other social media platforms distributing news in other European countries [47]. Confidence in news is much lower in other European countries than the Netherlands [48]. For example, 59% of people in the Netherlands trust the news, whereas in Italy and France the number is much lower (42% and 35%, respectively). In addition, Facebook is used more as a news source the latter two countries. In the Netherlands, 29% of people use Facebook as a news source, while in Italy and France the numbers are higher (51% and 41%, respectively) [48]. Another recommendation for follow-up research is to conduct the same research with different sources. In this study, we chose the most reliable source in the Netherlands, the NOS [40], and an unreliable source, Dailybuzzlive.nl [40], but it would be very interesting to also test different sources to increase external validity.

At the end of 2016, there was much commotion and media attention around the American presidential elections due to the relatively new phenomenon of online disinformation. Recently, it emerged that millions of Facebook users had seen extreme-right disinformation on the platform in the run-up to the European parliamentary elections [49]. And currently, the European Commission needed to respond to several alternative theories explaining the cause of the COVID-19 crisis because an increasing number of people believe fiction instead of facts [1]. As far as we know, this study was the first to show that source information has an effect on critical evaluation by Dutch Facebook users in the context of online disinformation. This means that critical evaluation can be encouraged when source information is provided, which might help to reduce the negative effects of online disinformation [50,51].

## Appendix

Multimedia Appendix 1 CONSORT-eHEALTH checklist (V 1.6.1).

## Abbreviations

ANCOVA: analysis of covariance
MANCOVA: multivariate analysis of covariance
NOS: Nederlands Omroep Stichting
PCA: principal component analysis
UNESCO: United Nations Educational, Scientific and Cultural Organization

## Data Availability

We will make the data available upon reasonable request.
